# Once fat was fat and that was that: our changing perspectives on adipose tissue

**DOI:** 10.5830/CVJA-2010-083

**Published:** 2011-06

**Authors:** WF Ferris, NJ Crowther

**Affiliations:** Division of Endocrinology, Department of Medicine, Faculty of Health Sciences, University of Stellenbosch, Stellenbosch, South Africa; Department of Chemical Pathology, National Health Laboratory Services, University of Witwatersrand Medical School, Johannesburg, South Africa

**Keywords:** adipocytes, obesity, cardiovascular disease, stem cells

## Abstract

**Abstract:**

Past civilisations saw excess body fat as a symbol of wealth and prosperity as the general population struggled with food shortages and famine. Nowadays it is recognised that obesity is associated with co-morbidities such as cardiovascular disease and diabetes. Our views on the roll of adipose tissue have also changed, from being solely a passive energy store, to an important endocrine organ that modulates metabolism, immunity and satiety. The relationship between increased visceral adiposity and obesity-related co-morbidities has lead to the recognition that variation in fat distribution contributes to ethnic differences in the prevalence of obesity-related diseases. Our current negative view of adipose tissue may change with the use of pluripotent adipose-derived stromal cells, which may lead to future autologous stem cell therapies for bone, muscle, cardiac and cartilage disorders. Here, we briefly review the concepts that adipose tissue is an endocrine organ, that differences in body fat distribution underline the aetiology of obesity-related co-morbidities, and the use of adipose-derived stem cells for future therapies.

## A changing view of adiposity through the ages

The incidence of obesity and obesity-related co-morbidities has risen dramatically in the last century. The latest global data shows that in 2004 cardiovascular disease was the primary cause of death, above infectious and parasitic diseases, with the majority of cases attributed to an unhealthy lifestyle. This includes over-nutrition.1 The increase in obesity has been accompanied by increased interest in fat and an abundance of research investigating the link between excessive adiposity and the associated pathologies. Currently there are over 130 000 research articles on obesity cited on PubMed and these publications show that our perception of the function of fat mass has changed considerably since the first entry cited from 1880. However, our knowledge of adiposity stretches back far beyond the 19th century. Although it is not known whether classical scholars recognised that adipose tissue is our major energy store, they did observe that excessive adiposity has negative health implications.

The Indian physician Sushruta (sixth century BCE) was probably the first to document a relationship between obesity and co-morbidities such as diabetes and heart disease. Not unlike today, he recommended exercise to remedy conditions that had arisen from a sedentary lifestyle and ‘pampering the belly’.2 Later in Europe, Hippocrates (460–377 BC) independently recognised the relationship between body composition, exercise and health, exemplified in his quote: ‘If we could give every individual the right amount of nourishment and exercise, not too little and not too much, we would have found the safest way to health’. In a time of scant medical knowledge, his insight extended further, beyond his contemporaries, to include the pathogenicity of obesity, in writing: ‘Repletion, carried to extremes, is perilous’ and ‘Corpulence is not only a disease in itself, but the harbinger of others’. He then subsequently noted that life expectancy was far shorter in the obese compared to lean individuals.3

Although the detrimental effects of obesity have therefore long been known, in the intervening millennia since Sushruta and Hippocrates, portliness was generally regarded as a symbol of affluence. This was primarily due to periodic food shortages and famine, which were only brought under control in the Western world in the last century yet still ravish the developing world today. This association between wealth and increased body mass was often reflected in the art of European masters such as Rubens (1577–1640) who depicted women with a full-bodied, hour-glass shape; a shape which was associated with opulence and fertility.4

By the 20th century, the use of intensive farming in conjunction with the mechanisation of the food industry helped to eradicate famine in the developed world. The increasing availability of highly palatable, high-energy foods and decreased levels of physical activity has lead to an increasing imbalance between energy input and expenditure in the general population. The consequence of this is a burgeoning of portliness and obesity. This rise in the prevalence of obesity is a global phenomenon, occurring in both the developed and the developing worlds. Data from the USA shows that in the period 1988–1994 the prevalence of obesity was 22.5%,5 and rose to 32.2% in the period 2003–2004.6 A meta-analysis of studies measuring prevalence of obesity in west African countries showed that the prevalence of obesity in urban areas rose from 7.0% in 1990–1994 to 15.0% by 2000–2004.7 Data from China demonstrated that the prevalence of overweight and obesity was 14.6% in 1992 and 21.8% in 2002.8 Similar trends have been reported around the world. The increasing prevalence of obesity in the developing world is compounded by the cultural view of obesity as being a positive attribute, signifying both health and wealth. This is particularly so in African nations,9 and is in stark contrast to the Western ideal, as portrayed in the mass media, of thin is beautiful!

## Central adiposity, ectopic fat deposition and obesity-related co-morbidities

‘Not all fat is created equal’ may be the new dogma in obesity research, with many studies reporting that the pathological effects of excessive adiposity are dependent not only on the quantity of fat, but on the distribution of the fat mass. The adipose tissue surrounding the major abdominal organs, the visceral fat, is thought to be the principal adipose depot involved in the aetiology of obesity-related disorders, with the subcutaneous fat depot playing a less prominent role.10 Closer scrutiny of adipocytes isolated from these two fat depots has corroborated this view and shown fundamental metabolic differences as well as a higher production from visceral adipocytes of adipose tissue-derived cytokines (adipokines), which may play an important role in the aetiology of many obesity-related diseases.11

It has been proposed that the rate of lipid uptake is greater in the subcutaneous than the visceral adipose tissue depot until the former site approaches its limit for lipid storage, when triglyceride uptake into the visceral depot predominates.12,13 Lipid accumulation in obesity promotes both adipocyte hyperplasia and hypertrophy,14,15 with storage mainly occurring in pre-existing adipocytes. As hypertrophy progresses, the storage capacity of the cells in subcutaneous adipose tissue becomes limiting and lipids that are not readily accumulated are shunted to the visceral stores. Excessive fat accumulation in the visceral stores leads to the secretion of free fatty acids into the portal vein, which, with the secretion of pro-inflammatory adipokines, leads to hepatic insulin resistance and aberrant accumulation of lipids in hepatocytes and the resultant hepatic steatosis.16

In obese individuals, the inadequate lipid storage capacity of the body’s adipose tissue depots leads to ectopic fat deposition not only in the liver but in other organs such as skeletal muscle and the insulin secreting β-cells of the pancreas. It has been suggested that this ectopic fat deposition may play an important role in the aetiology of both insulin resistance and β-cell failure.17 Furthermore, in obesity, increased fat deposition has also been noted peri-vascularly and peri-cardially and within myocytes.18 It has been suggested that this may contribute to vascular stiffness, cardiac dysfunction, hypertension, atherosclerosis and sodium retention, which are all characteristics of the cardiovascular disease observed in obese subjects.18

## Adipose tissue, a paracrine and endocrine organ

Adipose tissue is no longer just seen as a fat store, but is considered a true secretory tissue, with differences in secretion underpinning the greater pathogenicity of visceral than subcutaneous fat masses. Adipocytes are known to secrete pro-inflammatory cytokines such as TNFα and IL-6, which in conjunction with elevated free fatty acids (FFAs), promote insulin resistance.11 These cytokines are elevated in obesity and have been proposed to act in an autocrine loop, inhibiting the adipocyte hyperplastic response, which in turn leads to hypertrophy and further secretion of FFAs and pro-inflammatory cytokines.

Adipocytes produce a multitude of secreted peptides other than pro-inflammatory cytokines that have been linked to some of the obesity-related co-morbidities. Many of these molecules, such as plasminogen activation inhibitor 1 (PAI-1), angiotensinogen (AGT), monocyte chemo-attractant protein 1 (MCP-1) and resistin, have effects on vascular function. Plasminogen activation inhibitor 1 inhibits plasminogen activation and leads to fibrinolysis and a pro-thrombotic state.19,20 PAI-1 is secreted more by visceral than subcutaneous fat21 and is also a risk factor for coronary artery disease (CAD),22 whereas angiotensinogen has been implicated in the aetiology of hypertension and is upregulated in obesity,23,24 with production being higher in visceral fat.25 Furthermore, angiotensinogen is the precursor of angiotensin II of the vasoconstriction renin–angiotensin system and may be a causal agent for the hypertension seen during obesity.26

Monocyte chemo-attractant protein 1 is also secreted predominantly from the visceral depot, is overproduced during obesity and participates in the recruitment of macrophages and monocytes into the arterial cell wall. As this recruitment may lead to atherosclerosis, MCP-1 was measured in patients with or without CAD, and it was found to be elevated in the former group.27 Adipocytes also secrete resistin, which stimulates inflammatory cytokine production, as well as decreasing endothelial cell adhesion molecule (iCAM-1, vCAM-1, Ccl-2) production, which may promote atherosclerosis.28 The role of resistin in insulin resistance is still unclear.29,30

Adipose tissue also secretes other peptides that have effects peripherally and centrally. The most investigated of these is leptin, a satiety factor which was first characterised in a rodent model of monogenic obesity, the ob/ob mouse.31 Since the isolation and characterisation of leptin (from the Greek leptos: thin), adipose tissue has been viewed as a true endocrine organ. Leptin is secreted by adipocytes and modulates food intake by suppressing orexigenic peptides (Agouti-related peptide and neuropeptide Y) and upregulates anorexigenic peptides (corticotropin-releasing hormone and a-melanocyte stimulating hormone) in the brain.32 It also stimulates fatty acid oxidation and prevents lipid accumulation in adipose tissue.33,34 This forms a negative feedback mechanism, where increased fat mass produces more leptin, which reduces food intake, inhibiting further adipose expansion and limiting leptin expression. It was initially thought that this feedback loop could be used to inhibit food intake in the obese, but clinical trials of leptin analogues had little success, because endogenous leptin has since been found to be elevated in the obese, who often exhibit leptin resistance.35 The adipokine has since been attributed to being a signal for energy deficiency, rather than a signal to lose weight, as excessive weight loss will result in decreased leptin levels and a consequential increase in food intake.36,37

Since the characterisation of leptin, many other adipokines have been discovered, such as apelin, visfatin, chemerin and vaspin, with adiponectin being the most fully studied. Adiponectin is copiously secreted from mature adipocytes,38-40 with expression negatively correlating with body mass index (BMI).41,42 Consequently, lean subjects have high levels, whereas obese subjects have low plasma levels. Decreased expression of adiponectin is observed in a number of obesity-related co-morbidities such as type 2 diabetes,43,44 the metabolic syndrome,45,46 non-alcoholic steatohepatitis16 and CAD.47,48 It has also been found that the protein is anti-diabetic, increasing insulin sensitivity, glucose uptake and fat oxidation, as well as suppressing hepatic glucose output.49-51 The protein may also alter basal insulin secretion52 and modulate satiety, increasing food intake and suppressing energy expenditure when fasting, but surprisingly having opposite effects after refeeding.53 It is also anti-atherogenic47,54 and anti-inflammatory.55

Whereas adiponectin decreases during obesity, there are other glucose-lowering adipokines that correlate positively with BMI. Circulating apelin increases in obesity56 and has been show to lower glucose in normal and obese mice.57 Homozygous apelin knockout mice have severe heart failure in response to pressure overload and diminished heart contractility in aged mice,58 indicating a role for the adipokine in maintaining cardiac function. Visfatin is an adipokine that is predominantly expressed in visceral adipose tissue and has been attributed to having insulin-like properties,59 although this has since been disputed,60,61 and recently visfatin has been shown to have pro-inflammatory effects.62 Vaspin is a serine protease inhibitor and is reported to reduce expression of leptin, resistin and TNFα and improves insulin sensitivity.63,64

The recently discovered adipokine chemerin65,66 increases insulin sensitivity in 3T3-L1 adipocytes67 and is essential for normal adipocyte differentiation.65,66,68 However, it has also been shown to lower glucose tolerance in murine models of obesity/diabetes69 and to cause insulin resistance in human skeletal muscle cells, where it was also observed to be pro-inflammatory.70 Consequently adipose tissue secretes both pro- and anti-inflammatory cytokines which modulate metabolism by altering insulin resistance. Generally, pro-inflammatory cytokine production increases and anti-inflammatory expression decreases during insulin resistance and obesity.

## Obesity and cardiovascular disease in Africa

Studies have shown that the prevalence of both cardiovascular disease (CVD)71 and obesity7 is rising in Africa. Although it is not certain that these two findings are linked, the observation that CVD is more common in obese Africans72 supports this premise. This recent rise in the prevalence of obesity in Africa is attributed to increased urbanisation and the associated ease of access to a more westernised, calorie-dense diet.73

Within Africa, the prevalence of CVD and its risk factors differs across the various resident population groups. Accordingly, mortality due to heart disease is higher in the Asian-Indian and European ethnic groups of South Africa when compared to the indigenous black African population.74 Fasting serum cholesterol and triglyceride levels are higher in Asian-Indian than African subjects,75 with type 2 diabetes being more prevalent in the former population group.76 The reasons for these ethnic differences in disease prevalence rates and cardiovascular risk factors are not fully understood, although it has been suggested that the higher abdominal fat mass observed in Asian-Indian and European compared to African subjects may be involved.77

It is, however, of note that African subjects tend to be more insulin resistant than Europeans78,79 even though they have less visceral adiposity. This would suggest either that visceral fat in African compared to European subjects has a greater ability to reduce insulin sensitivity, or that visceral adiposity is not involved in determining the level of whole-body insulin sensitivity in the African population. The latter hypothesis is unlikely since it has been shown that waist circumference, independently of BMI, is a determinant of insulin sensitivity in this population group.80 It is also possible that subcutaneous abdominal fat may play a more prominent role in determining whole-body insulin sensitivity in African than European females, as has been observed in a previous study.79

Previous investigators have suggested that obesity in African subjects is benign. This hypothesis was based on reports that blood pressure, glucose and lipid levels were not elevated in obese compared to lean African females.81 However, this hypothesis is challenged by data showing that there is a higher prevalence of CVD in obese compared to non-obese African subjects.72 Furthermore, it must be noted that these studies81 did not take into account body fat distribution, which is a major contributing factor to the pathogenesis of obesity-related disorders. It is also of interest to note that the African countries with the highest prevalence of obesity have the highest prevalence of obesity-related disorders, such as type 2 diabetes.82

## Adiposity and insulin resistance as a biological advantage

Obesity has many negative connotations with regard to health. It is associated with an increased risk of many diseases, ranging from asthma to cancer. However, body fat does have an important physiological role, including the maintenance of body temperature and triglyceride storage. It also acts as an endocrine modulator of insulin sensitivity and appetite. The negative effects of adiposity on insulin sensitivity are often viewed as purely pathological. However, insulin resistance has been proposed to have an important biological role. It is now thought that insulin resistance is a normal physiological response to obesity to slow down triglyceride deposition in adipose tissue.83 Studies have indeed shown that insulin resistance may protect against weight gain.84,85 Furthermore, the biological adaptation of insulin resistance has been proposed as advantageous in prehistory, during times of feast and famine. The ability to readily store energy as fat would be beneficial until excessive adiposity would limit the capability of our ancestors to hunt and escape predation. Thus, insulin resistance would act to limit the rate of fat deposition. It is therefore possible that insulin resistance evolved to limit fat deposition in a period of human evolutionary history when excessive caloric intake was not a common occurrence. In modern times however, access to calorie-dense foods is not limited and this homeostatic mechanism for limiting excessive weight gain has been overpowered by new environmental conditions in which famine has been replaced by feast.

Adipose tissue may play an important role in modulating immunity. Adipocytes secrete a wide range of different cytokines that have both pro- and anti-inflammatory properties. Also, lymph nodes are normally found within adipose tissue depots and studies have demonstrated a strong interrelationship between these two tissue types. Therefore, the cells of the lymph node are supplied with specialised free fatty acids by the perinodal adipocytes, and dendritic cells from the lymph nodes are able to modulate lipolysis of the surrounding adipose tissue.86 Furthermore, the adipokine, leptin has been shown to have effects on immune system functionality. Subjects with a leptin gene mutation have very low serum leptin levels and reduced numbers of CD_4_^+^ T cells and low T-cell proliferation rates. All these defects are normalised by administration of exogenous leptin.87

## Fat as a source of stem cells

Our perception of adiposity has recently changed again. In addition to being an energy store, a major protagonist in the development of insulin resistance and a modulator of satiety, adipose tissue has been found to be an abundant store of stem cells. Adipose tissue may therefore be seen more positively, given that these cells may be used to treat a multitude of diseases.

Originally, Young *et al*.88 isolated stem cells by digesting the connective tissue in fat and cultured the liberated cells, which they labelled the stromal vascular fraction (SVF). This was an unpurified population containing stromal cells, endothelial progenitor cells, fibroblasts and haematopoietic stem cells,89 which were used to produce neo-vascular cells. The multipotential mesenchymal precursor cells that are harboured within the SVF may not only be differentiated into adipocytes,90-92 but also bone-forming osteoblasts,90,93,94 muscle myoblasts,93,95 cardiomyocytes96 and cartilage-forming chondrocytes.90,93 Consequently, there is considerable interest in these adipose-derived stromal cells (ADSCs)93,94 for regenerative medicine. This is not only for the replacement of damaged fat,97 bone,98-100 muscle101 and cartilage, 102 for it has been found that ADSCs also secrete cytokines, such as VEGF, HGF and SDF-19,103,104 which stimulate angiogenesis. These cells may therefore be used to treat ischaemic disease,105 such as fibrosis and osteoradionecrosis, which are late complications of radiotherapy.106 It has also been found that the growth factors that ADSCs secrete stimulate fibroblast and keratinocyte growth and therefore ADSCs have been used to aid skin repair.107 Unlike bone marrow-derived stromal cells (BMSCs), a prominent redeeming feature of ADSCs is their ease of isolation.108

ADSCs and fat transplantation have been successfully used after trauma and surgical resection such as mastectomy,109,110 where ADSCs help to abrogate problems with angiogenesis and the long-term viability of grafts.111-113 ADSCs have also been used to treat lipodystrophy,114 which has become common due to side effects of antiretroviral therapies (ART) in HIV-positive patients.115,116 These ADSCs are expanded in number *in vitro* and differentiated into mature adipocytes using a cocktail including insulin, the cAMP inducer IBMX, a PPARg agonist indomethecin and a low concentration of a glucocorticoid such as dexamethasone.117,118 The use of different cocktails enables ADSCs to be differentiated into osteoblasts, myocytes or chondrocytes.

Lee *et al*.119 was the first to demonstrate that ADSCs could be differentiated into bone-forming osteoblasts and these cells were used to heal critically sized calvarial defects in mice. In a direct comparison during this investigation, ADSCs were found to have the same efficacy as BMSCs. It was established, using genetic analysis that 96% of the new bone was from the female donor rather than from the male recipient.120 As both adults and children over the age of two years are unable to correct large cranial defects due to inadequate ossification, this application has direct relevance in man and was first used to correct a 120-cm^2^ defect in a seven-year-old girl with a severe head injury.121

The differentiation of ADSCs into myocytes is relatively inefficient and gives a low yield and low reproducibility.89 Glucocorticoids and 5% horse serum are used to supplement the growth media to stimulate the fusion of cells to form multi-nucleated myotubes which express myocyte markers.90,93,122 Although *in vitro* differentiation is far from optimal, these cells have been used to correct defects in the tibialis anterior muscle in a mouse model for Duchennes’s muscular dystrophy.

The differentiation of ADSCs into chondrocytes is also inefficient. Insulin, TGFβ1 and ascorbic acid122,123 are used to stimulate chondogenesis in ADSCs, which takes two weeks, but unfortunately the yield is far less than when using BMSCs.123 As cartilage repair *in vivo* is often difficult and slow, the use of ADSCs to treat traumatised and arthritic joints and to aid joint reconstruction still warrants further research102 and promises to improve therapy for cartilage repair in the future.

Adult mesenchymal stem cells isolated from the adipose tissue of rabbits are able to differentiate into cardiomyocytes when treated with 5-azacytidine.96 This process has also been observed in human ADSCs cultured in the presence of dimethylsulfoxide.124 Furthermore, such cells were used to improve cardiac function and increase survival rate in a rodent model of myocardial infarction.124 Similar results were obtained in experiments in which undifferentiated ADSCs were transplanted into rodent125,126 and porcine127 infarcted hearts. These data suggest that at least in non-human models of myocardial infarction, ADSCs may be used to repair damaged cardiac tissue, although their utility in humans is still not known and requires further investigation.

## Fat and the future

The future certainly looks secure for fat. The prevalence of obesity in the developing world shows no sign of abating, although recent data from the USA shows evidence of plateauing. 128 The rising levels of obesity in Africa were expected to result in an increase in the prevalence of obesity-related disorders, which seems to be the case.71,129 Africa is also the centre of an HIV/AIDS epidemic and is therefore suffering a double burden of communicable and non-communicable diseases. Studies have shown that HIV infection and ART can both lead to cardiovascular disease130 and this will further enhance the current epidemic of obesity-related diseases on the African continent. Consequently, the use of ART has converted our view of HIV infection from a certain death sentence to a chronic disease, and this is leading to the development of health service infrastructures that can be used for HIV diagnosis, ART roll out and patient follow up. Such infrastructure could also be utilised for the diagnosis and monitoring of non-communicable diseases in both HIV-positive and HIV-negative subjects.131

There are a number of interesting aspects of obesity in African populations that deserve continued investigation. The more diabetogenic than atherogenic nature of adiposity in African compared to European subjects is not well understood and unravelling the molecular mechanisms involved in such ethnic differences may well uncover new aetiological pathways of obesity-related diseases. The difference in body fat distribution between population groups is also worthy of further study, particularly as African subjects have less visceral fat than BMI-matched Europids, and yet are more insulin resistant.77-79 The use of high-throughput gene-screening technology, which has yielded important information on the polygenic nature of obesity via genome-wide association studies132 should therefore be used in African populations to determine the genetic input to adiposity and body fat distribution. It is possible that ethnic differences in insulin sensitivity and the prevalence of obesity-related disorders are due to differences in the secretory output of adipocytes. The comparison of adipocyte secretomes across population groups using the new technologies developed for the analysis of complex mixtures of bioactive molecules133 may therefore be very worthwhile.

The future of the use of adipose-derived stromal cells (ADSCs) for the treatment of human disease looks very promising. Such cells have already been used to correct cranial defects in humans,119 and preliminary studies in man to rectify cardiovascular134,135 and soft tissue136-138 defects hold hope for the future use of ADSCs in the treatment of muscle and cartilage defects and heart infarcts. However, before this becomes a reality, there are a number of technical problems that need to be overcome. The methods used for the large-scale isolation of ADSCs and their efficient conversion into the correct cell phenotype must be improved and standardised. Also, the long-term safety of the use of these cells in humans must be explored, initially by the development of the appropriate animal models. Stem-cell therapy is already available for the treatment of haematological malignancies in specialised medical centres within Africa139 and therefore it is feasible that the therapeutic use of ADSCs may also become a reality for this continent.

## Conclusion

Our view of adipose tissue has changed over time. Additional information has led us to confirm that fat is not only a store of energy, but when in excess, it is the instigator of obesity-related co-morbidities. The characterisation of adipokines has led to the realisation that adipose tissue is a true endocrine organ, and the isolation and use of ADSCs has led to hope for future therapeutic treatments of degenerative diseases of fat, bone, muscle and cartilage. Once fat was just fat, but it is now much more than that.

## References

[1] Mathers CD, Boerma T, Ma FD (2009). Global and regional causes of death.. Br Med Bull.

[2] Tipton CM (2008). Historical perspective: the antiquity of exercise, exercise physiology and the exercise prescription for health.. Wld Rev Nutr Diet.

[3] Cheng TO (2001). Hippocrates and cardiology.. Am Heart J.

[4] Woodhouse R (2008). Obesity in art: a brief overview.. Front Horm Res.

[5] Flegal KM, Carroll MD, Kuczmarski RJ, Johnson CL (1998). Overweight and obesity in the United States: prevalence and trends, 1960–1994.. Int J Obes Relat Metab Disord.

[6] Ogden CL, Carroll MD, Curtin LR, McDowell MA, Tabak CJ, Flegal KM (2006). Prevalence of overweight and obesity in the United States, 1999–2004.. J Am Med Assoc.

[7] Abubakari AR, Lauder W, Agyemang C, Jones M, Kirk A, Bhopal RS (2008). Prevalence and time trends in obesity among adult West African populations: a meta-analysis.. Obes Rev.

[8] Wang Y, Mi J, Shan XY, Wang QJ, Ge KY (2007). Is China facing an obesity epidemic and the consequences? The trends in obesity and chronic disease in China.. Int J Obes (Lond).

[9] Holdsworth M, Gartner A, Landais E, Maire B, Delpeuch F (2004). Perceptions of healthy and desirable body size in urban Senegalese women.. Int J Obes Relat Metab Disord.

[10] Kissebah AH, Krakower GR (1994). Regional adiposity and morbidity.. Physiol Rev.

[11] Hamdy O, Porramatikul S, Al Ozairi E (2006). Metabolic obesity: the paradox between visceral and subcutaneous fat.. Curr Diabetes Rev.

[12] Frayn KN (2000). Visceral fat and insulin resistance – causative or correlative?. Br J Nutr.

[13] Sniderman AD, Bhopal R, Prabhakaran D, Sarrafzadegan N, Tchernof A (2007). Why might South Asians be so susceptible to central obesity and its atherogenic consequences? The adipose tissue overflow hypothesis.. J Epidemiol.

[14] Sjostrom L, William-Olsson T (1981). Prospective studies on adipose tissue development in man.. Int J Obes.

[15] Couillard C, Mauriege P, Imbeault P, Prud’homme D, Nadeau A, Tremblay A (2000). Hyperleptinemia is more closely associated with adipose cell hypertrophy than with adipose tissue hyperplasia.. Int J Obes Relat Metab Disord.

[16] Mendez-Sanchez N, Arrese M, Zamora-Valdes D, Uribe M (2007). Current concepts in the pathogenesis of nonalcoholic fatty liver disease.. Liver Int.

[17] Ravussin E, Smith SR (2002). Increased fat intake, impaired fat oxidation, and failure of fat cell proliferation result in ectopic fat storage, insulin resistance, and type 2 diabetes mellitus.. Ann N Y Acad Sci.

[18] Montani JP, Carroll JF, Dwyer TM, Antic V, Yang Z, Dulloo AG (2004). Ectopic fat storage in heart, blood vessels and kidneys in the pathogenesis of cardiovascular diseases.. Int J Obes Relat Metab Disord.

[19] Alessi MC, Poggi M, Juhan-Vague I (2007). Plasminogen activator inhibitor-1, adipose tissue and insulin resistance.. Curr Opin Lipidol.

[20] Mertens I, Verrijken A, Michiels JJ, Van der PM, Ruige JB, Van Gaal LF (2006). Among inflammation and coagulation markers, PAI-1 is a true component of the metabolic syndrome.. Int J Obes (Lond).

[21] Gottschling-Zeller H, Birgel M, Rohrig K, Hauner H (2000). Effect of tumor necrosis factor alpha and transforming growth factor beta 1 on plasminogen activator inhibitor-1 secretion from subcutaneous and omental human fat cells in suspension culture.. Metabolism.

[22] Juhan-Vague I, Vague P (1991). Hypofibrinolysis and insulin-resistance.. Diabetes Metab.

[23] Umemura S, Nyui N, Tamura K, Hibi K, Yamaguchi S, Nakamaru M (1997). Plasma angiotensinogen concentrations in obese patients.. Am J Hypertens.

[24] Van H V, Ariapart P, Hoffstedt J, Lundkvist I, Bringman S, Arner P (2000). Increased adipose angiotensinogen gene expression in human obesity.. Obes Res.

[25] Giacchetti G, Faloia E, Sardu C, Camilloni MA, Mariniello B, Gatti C (2000). Gene expression of angiotensinogen in adipose tissue of obese patients.. Int J Obes Relat Metab Disord.

[26] Massiera F, Bloch-Faure M, Ceiler D, Murakami K, Fukamizu A, Gasc JM (2001). Adipose angiotensinogen is involved in adipose tissue growth and blood pressure regulation.. Fed Am Soc Exp Biol J.

[27] Martinovic I, Abegunewardene N, Seul M, Vosseler M, Horstick G, Buerke M (2005). Elevated monocyte chemoattractant protein-1 serum levels in patients at risk for coronary artery disease.. Circ J.

[28] Verma S, Li SH, Wang CH, Fedak PW, Li RK, Weisel RD (2003). Resistin promotes endothelial cell activation: further evidence of adipokineendothelial interaction.. Circulation.

[29] Savage DB, Sewter CP, Klenk ES, Segal DG, Vidal-Puig A, Considine RV (2001). Resistin / Fizz3 expression in relation to obesity and peroxisome proliferator-activated receptor-gamma action in humans.. Diabetes.

[30] Utzschneider KM, Carr DB, Tong J, Wallace TM, Hull RL, Zraika S (2005). Resistin is not associated with insulin sensitivity or the metabolic syndrome in humans.. Diabetologia.

[31] Zhang Y, Proenca R, Maffei M, Barone M, Leopold L, Friedman JM (1994). Positional cloning of the mouse obese gene and its human homologue.. Nature.

[32] Ahima RS (2008). Revisiting leptin’s role in obesity and weight loss.. J Clin Invest.

[33] Minokoshi Y, Kim YB, Peroni OD, Fryer LG, Muller C, Carling D (2002). Leptin stimulates fatty-acid oxidation by activating AMP-activated protein kinase.. Nature.

[34] Muoio DM, Dohm GL, Fiedorek FT, Tapscott EB, Coleman RA (1997). Leptin directly alters lipid partitioning in skeletal muscle.. Diabetes.

[35] Myers MG, Cowley MA, Munzberg H (2008). Mechanisms of leptin action and leptin resistance.. A Rev Physiol.

[36] Badman MK, Flier JS (2007). The adipocyte as an active participant in energy balance and metabolism.. Gastroenterology.

[37] Kershaw EE, Flier JS (2004). Adipose tissue as an endocrine organ.. J Clin Endocrinol Metab.

[38] Maeda K, Okubo K, Shimomura I, Funahashi T, Matsuzawa Y, Matsubara K (1996). cDNA cloning and expression of a novel adipose specific collagen-like factor, apM1 (AdiPose Most abundant Gene transcript 1).. Biochem Biophys Res Commun.

[39] Scherer PE, Williams S, Fogliano M, Baldini G, Lodish HF (1995). A novel serum protein similar to C1q, produced exclusively in adipocytes.. J Biol Chem.

[40] Halleux CM, Takahashi M, Delporte ML, Detry R, Funahashi T, Matsuzawa Y (2001). Secretion of adiponectin and regulation of apM1 gene expression in human visceral adipose tissue.. Biochem Biophys Res Commun.

[41] Arita Y, Kihara S, Ouchi N, Takahashi M, Maeda K, Miyagawa J (1999). Paradoxical decrease of an adipose-specific protein, adiponectin, in obesity.. Biochem Biophys Res Commun.

[42] Brichard SM, Delporte ML, Lambert M (2003). Adipocytokines in anorexia nervosa: a review focusing on leptin and adiponectin.. Horm Metab Res.

[43] Weyer C, Funahashi T, Tanaka S, Hotta K, Matsuzawa Y, Pratley RE (2001). Hypoadiponectinemia in obesity and type 2 diabetes: close association with insulin resistance and hyperinsulinemia.. J Clin Endocrinol Metab.

[44] Hotta K, Funahashi T, Bodkin NL, Ortmeyer HK, Arita Y, Hansen BC (2001). Circulating concentrations of the adipocyte protein adiponectin are decreased in parallel with reduced insulin sensitivity during the progression to type 2 diabetes in rhesus monkeys.. Diabetes.

[45] Gilardini L, McTernan PG, Girola A, da Silva NF, Alberti L, Kumar S (2006). Adiponectin is a candidate marker of metabolic syndrome in obese children and adolescents.. Atherosclerosis.

[46] Mohan V, Deepa R, Pradeepa R, Vimaleswaran KS, Mohan A, Velmurugan K (2005). Association of low adiponectin levels with the metabolic syndrome – the Chennai Urban Rural Epidemiology Study (CURES-4).. Metabolism.

[47] Ouchi N, Kihara S, Arita Y, Maeda K, Kuriyama H, Okamoto Y (1999). Novel modulator for endothelial adhesion molecules: adipocyte-derived plasma protein adiponectin.. Circulation.

[48] Wang ZV, Scherer PE (2008). Adiponectin, cardiovascular function, and hypertension.. Hypertension.

[49] Fruebis J, Tsao TS, Javorschi S, Ebbets-Reed D, Erickson MR, Yen FT (2001). Proteolytic cleavage product of 30-kDa adipocyte complementrelated protein increases fatty acid oxidation in muscle and causes weight loss in mice.. Proc Natl Acad Sci USA.

[50] Berg AH, Combs TP, Du X, Brownlee M, Scherer PE (2001). The adipocytesecreted protein Acrp30 enhances hepatic insulin action.. Nat Med.

[51] Yamauchi T, Kamon J, Waki H, Terauchi Y, Kubota N, Hara K (2001). The fat-derived hormone adiponectin reverses insulin resistance associated with both lipoatrophy and obesity.. Nat Med.

[52] Okamoto M, Ohara-Imaizumi M, Kubota N, Hashimoto S, Eto K, Kanno T (2008). Adiponectin induces insulin secretion in vitro and in vivo at a low glucose concentration.. Diabetologia.

[53] Kubota N, Yano W, Kubota T, Yamauchi T, Itoh S, Kumagai H (2007). Adiponectin stimulates AMP-activated protein kinase in the hypothalamus and increases food intake.. Cell Metab.

[54] Beauloye V, Zech F, Tran HT, Clapuyt P, Maes M, Brichard SM (2007). Determinants of early atherosclerosis in obese children and adolescents.. J Clin Endocrinol Metab.

[55] Kato H, Kashiwagi H, Shiraga M, Tadokoro S, Kamae T, Ujiie H (2006). Adiponectin acts as an endogenous antithrombotic factor.. Arterioscler Thromb Vasc Biol.

[56] Heinonen MV, Purhonen AK, Miettinen P, Paakkonen M, Pirinen E, Alhava E (2005). Apelin, orexin-A and leptin plasma levels in morbid obesity and effect of gastric banding.. Regul Pept.

[57] Dray C, Knauf C, Daviaud D, Waget A, Boucher J, Buleon M (2008). Apelin stimulates glucose utilization in normal and obese insulinresistant mice.. Cell Metab.

[58] Kuba K, Zhang L, Imai Y, Arab S, Chen M, Maekawa Y (2007). Impaired heart contractility in Apelin gene-deficient mice associated with aging and pressure overload.. Circ Res.

[59] Fukuhara A, Matsuda M, Nishizawa M, Segawa K, Tanaka M, Kishimoto K (2005). Visfatin: a protein secreted by visceral fat that mimics the effects of insulin.. Science.

[60] Berndt J, Kloting N, Kralisch S, Kovacs P, Fasshauer M, Schon MR (2005). Plasma visfatin concentrations and fat depot-specific mRNA expression in humans.. Diabetes.

[61] Varma V, Yao-Borengasser A, Rasouli N, Bodles AM, Phanavanh B, Lee MJ (2007). Human visfatin expression: relationship to insulin sensitivity, intramyocellular lipids, and inflammation.. J Clin Endocrinol Metab.

[62] Garten A, Petzold S, Korner A, Imai S, Kiess W (2009). Nampt: linking NAD biology, metabolism and cancer.. Trends Endocrinol Metab.

[63] Hida K, Wada J, Eguchi J, Zhang H, Baba M, Seida A (2005). Visceral adipose tissue-derived serine protease inhibitor: a unique insulin-sensitizing adipocytokine in obesity.. Proc Natl Acad Sci USA.

[64] Youn BS, Kloting N, Kratzsch J, Lee N, Park JW, Song ES (2008). Serum vaspin concentrations in human obesity and type 2 diabetes.. Diabetes.

[65] Bozaoglu K, Bolton K, McMillan J, Zimmet P, Jowett J, Collier G (2007). Chemerin is a novel adipokine associated with obesity and metabolic syndrome.. Endocrinology.

[66] Goralski KB, McCarthy TC, Hanniman EA, Zabel BA, Butcher EC, Parlee SD (2007). Chemerin, a novel adipokine that regulates adipogenesis and adipocyte metabolism.. J Biol Chem.

[67] Takahashi M, Takahashi Y, Takahashi K, Zolotaryov FN, Hong KS, Kitazawa R (2008). Chemerin enhances insulin signaling and potentiates insulin-stimulated glucose uptake in 3T3-L1 adipocytes.. FEBS Lett.

[68] Roh SG, Song SH, Choi KC, Katoh K, Wittamer V, Parmentier M (2007). Chemerin – a new adipokine that modulates adipogenesis via its own receptor.. Biochem Biophys Res Commun.

[69] Ernst MC, Issa M, Goralski KB, Sinal CJ (2010). Chemerin exacerbates glucose intolerance in mouse models of obesity and diabetes.. Endocrinology.

[70] Sell H, Laurencikiene J, Taube A, Eckardt K, Cramer A, Horrighs A (2009). Chemerin is a novel adipocyte-derived factor inducing insulin resistance in primary human skeletal muscle cells.. Diabetes.

[71] Akinboboye O, Idris O, Akinboboye O, Akinkugbe O (2003). Trends in coronary artery disease and associated risk factors in sub-Saharan Africans.. J Hum Hypertens.

[72] Tibazarwa K, Ntyintyane L, Sliwa K, Gerntholtz T, Carrington M, Wilkinson D (2009). A time bomb of cardiovascular risk factors in South Africa: results from the Heart of Soweto Study ‘Heart Awareness Days’.. Int J Cardiol.

[73] Yusuf S, Reddy S, Ounpuu S, Anand S (2001). Global burden of cardiovascular diseases: part I: general considerations, the epidemiologic transition, risk factors, and impact of urbanization.. Circulation.

[74] Walker AR, Sareli P (1997). Coronary heart disease: outlook for Africa.. J R Soc Med.

[75] Ferris WF, Naran NH, Crowther NJ, Rheeder P, van der ML, Chetty N (2005). The relationship between insulin sensitivity and serum adiponectin levels in three population groups.. Horm Metab Res.

[76] Mbanya JC, Motala AA, Sobngwi E, Assah FK, Enoru ST (2010). Diabetes in sub-Saharan Africa.. Lancet.

[77] Ali AT, Crowther NJ (2005). Body fat distribution and insulin resistance.. S Afr Med J.

[78] Van der Merwe M-T, Crowther NJ, Schlaphoff GP, Gray IP, Joffe BI, Lönnroth PN (2000). Evidence for insulin resistance in black women from South Africa.. Int J Obes.

[79] Goedecke JH, Levitt NS, Lambert EV, Utzschneider KM, Faulenbach MV, Dave JA (2009). Differential effects of abdominal adipose tissue distribution on insulin sensitivity in black and white South African women.. Obesity.

[80] Crowther NJ, Ferris WF, Ojwang PJ, Rheeder P (2006). The effect of abdominal obesity on insulin sensitivity and serum lipid and cytokine concentrations in African women.. Clin Endocrinol (Oxf).

[81] Walker AR, Walker BF, Manetsi B, Tsotetsi NG, Walker AJ (1990). Obesity in black women in Soweto, South Africa: minimal effects on hypertension, hyperlipidaemia and hyperglycaemia.. J R Soc Hlth.

[82] Ali AT, Crowther NJ (2010). The relationship between national socio-economic variables and obesity prevalence rates in Africa and Europe.. J Endocrinol Metab Diabetes S Afr.

[83] Eckel RH (1992). Insulin resistance: an adaptation for weight maintenance.. Lancet.

[84] Swinburn BA, Nyomba BL, Saad MF, Zurlo F, Raz I, Knowler WC (1991). Insulin resistance associated with lower rates of weight gain in Pima Indians.. J Clin Invest.

[85] Frayn KN, Humphreys SM, Coppack SW (1996). Net carbon flux across subcutaneous adipose tissue after a standard meal in normal-weight and insulin-resistant obese subjects.. Int J Obes Relat Metab Disord.

[86] Pond CM (2005). Adipose tissue and the immune system.. Prostaglandins Leukot Essent Fatty Acids.

[87] Farooqi IS, Matarese G, Lord GM, Keogh JM, Lawrence E, Agwu C (2002). Beneficial effects of leptin on obesity, T cell hyporesponsiveness, and neuroendocrine/metabolic dysfunction of human congenital leptin deficiency.. J Clin Invest.

[88] Young C, Jarrell BE, Hoying JB, Williams SK (1992). A porcine model for adipose tissue-derived endothelial cell transplantation.. Cell Transpl.

[89] Tholpady SS, Llull R, Ogle RC, Rubin JP, Futrell JW, Katz AJ (2006). Adipose tissue: stem cells and beyond.. Clin Plast Surg.

[90] Zuk PA, Zhu M, Ashjian P, De Ugarte DA, Huang JI, Mizuno H (2002). Human adipose tissue is a source of multipotent stem cells.. Mol Biol Cell.

[91] Wickham MQ, Erickson GR, Gimble JM, Vail TP, Guilak F (2003). Multipotent stromal cells derived from the infrapatellar fat pad of the knee.. Clin Orthop Relat Res.

[92] Gimble J, Guilak F (2003). Adipose-derived adult stem cells: isolation, characterization, and differentiation potential.. Cytotherapy.

[93] Zuk PA, Zhu M, Mizuno H, Huang J, Futrell JW, Katz AJ (2001). Multilineage cells from human adipose tissue: implications for cellbased therapies.. Tissue Eng.

[94] Gronthos S, Franklin DM, Leddy HA, Robey PG, Storms RW, Gimble JM (2001). Surface protein characterization of human adipose tissue-derived stromal cells.. J Cell Physiol.

[95] Gimble JM, Guilak F (2003). Differentiation potential of adipose derived adult stem (ADAS) cells.. Curr Top Dev Biol.

[96] Rangappa S, Fen C, Lee EH, Bongso A, Sim EK (2003). Transformation of adult mesenchymal stem cells isolated from the fatty tissue into cardiomyocytes.. Ann Thorac Surg.

[97] Sommer B, Sattler G (2000). Current concepts of fat graft survival: histology of aspirated adipose tissue and review of the literature.. Dermatol Surg.

[98] Kwan MD, Slater BJ, Wan DC, Longaker MT (2008). Cell-based therapies for skeletal regenerative medicine.. Hum Mol Genet.

[99] Slater BJ, Kwan MD, Gupta DM, Panetta NJ, Longaker MT (2008). Mesenchymal cells for skeletal tissue engineering.. Expert Opin Biol Ther.

[100] Hoogendoorn RJ, Lu ZF, Kroeze RJ, Bank RA, Wuisman PI, Helder MN (2008). Adipose stem cells for intervertebral disc regeneration: current status and concepts for the future.. J Cell Mol Med.

[101] Smaldone MC, Chen ML, Chancellor MB (2009). Stem cell therapy for urethral sphincter regeneration.. Minerva Urol Nefrol.

[102] Nathan S, Das DS, Thambyah A, Fen C, Goh J, Lee EH (2003). Cell-based therapy in the repair of osteochondral defects: a novel use for adipose tissue.. Tissue Eng.

[103] Rehman J, Traktuev D, Li J, Merfeld-Clauss S, Temm-Grove CJ, Bovenkerk JE (2004). Secretion of angiogenic and antiapoptotic factors by human adipose stromal cells.. Circulation.

[104] Cao Y, Sun Z, Liao L, Meng Y, Han Q, Zhao RC (2005). Human adipose tissue-derived stem cells differentiate into endothelial cells *in vitro* and improve postnatal neovascularization *in vivo*.. Biochem Biophys Res Commun.

[105] Murohara T, Shintani S, Kondo K (2009). Autologous adipose-derived regenerative cells for therapeutic angiogenesis.. Curr Pharm Des.

[106] Rigotti G, Marchi A, Galie M, Baroni G, Benati D, Krampera M (2007). Clinical treatment of radiotherapy tissue damage by lipoaspirate transplant: a healing process mediated by adipose-derived adult stem cells.. Plast Reconstr Surg.

[107] Kim WS, Park BS, Sung JH (2009). The wound-healing and antioxidant effects of adipose-derived stem cells.. Expert Opin Biol Ther.

[108] Kern S, Eichler H, Stoeve J, Kluter H, Bieback K (2006). Comparative analysis of mesenchymal stem cells from bone marrow, umbilical cord blood, or adipose tissue.. Stem Cells.

[109] Brey EM, Patrick CW (2000). Tissue engineering applied to reconstructive surgery.. IEEE Eng Med Biol Mag.

[110] Stosich MS, Mao JJ (2007). Adipose tissue engineering from human adult stem cells: clinical implications in plastic and reconstructive surgery.. Plast Reconstr Surg.

[111] Parker AM, Katz AJ (2006). Adipose-derived stem cells for the regeneration of damaged tissues.. Expert Opin Biol Ther.

[112] Stosich MS, Bastian B, Marion NW, Clark PA, Reilly G, Mao JJ (2007). Vascularized adipose tissue grafts from human mesenchymal stem cells with bioactive cues and microchannel conduits.. Tissue Eng.

[113] Halberstadt C, Austin C, Rowley J, Culberson C, Loebsack A, Wyatt S (2002). A hydrogel material for plastic and reconstructive applications injected into the subcutaneous space of a sheep.. Tissue Eng.

[114] Gavrilova O, Marcus-Samuels B, Graham D, Kim JK, Shulman GI, Castle AL (2000). Surgical implantation of adipose tissue reverses diabetes in lipoatrophic mice.. J Clin Invest.

[115] Carr A, Samaras K, Burton S, Law M, Freund J, Chisholm DJ (1998). A syndrome of peripheral lipodystrophy, hyperlipidaemia and insulin resistance in patients receiving HIV protease inhibitors.. AIDS.

[116] Davison SP, Timpone J, Hannan CM (2007). Surgical algorithm for management of HIV lipodystrophy.. Plast Reconstr Surg.

[117] Aust L, Devlin B, Foster SJ, Halvorsen YD, Hicok K, du LT (2004). Yield of human adipose-derived adult stem cells from liposuction aspirates.. Cytotherapy.

[118] Kim DH, Je CM, Sin JY, Jung JS (2003). Effect of partial hepatectomy on in vivo engraftment after intravenous administration of human adipose tissue stromal cells in mouse.. Microsurgery.

[119] Lee JA, Parrett BM, Conejero JA, Laser J, Chen J, Kogon AJ (2003). Biological alchemy: engineering bone and fat from fat-derived stem cells.. Ann Plast Surg.

[120] Cowan CM, Shi YY, Aalami OO, Chou YF, Mari C, Thomas R (2004). Adipose-derived adult stromal cells heal critical-size mouse calvarial defects.. Nat Biotechnol.

[121] Lendeckel S, Jodicke A, Christophis P, Heidinger K, Wolff J, Fraser JK (2004). Autologous stem cells (adipose) and fibrin glue used to treat widespread traumatic calvarial defects: case report.. J Craniomaxillofac Surg.

[122] Gimble JM (2003). Adipose tissue-derived therapeutics.. Expert Opin Biol Ther.

[123] Winter A, Breit S, Parsch D, Benz K, Steck E, Hauner H (2003). Cartilage-like gene expression in differentiated human stem cell spheroids: a comparison of bone marrow-derived and adipose tissue-derived stromal cells.. Arthritis Rheum.

[124] Okura H, Matsuyama A, Lee CM, Saga A, Kakuta-Yamamoto A, Nagao A (2010). Cardiomyoblast-like cells differentiated from human adipose tissue-derived mesenchymal stem cells improve left ventricular dysfunction and survival in a rat myocardial infarction model.. Tissue Eng Part C Meth.

[125] Miyahara Y, Nagaya N, Kataoka M, Yanagawa B, Tanaka K, Hao H (2006). Monolayered mesenchymal stem cells repair scarred myocardium after myocardial infarction.. Nat Med.

[126] Schenke-Layland K, Strem BM, Jordan MC, Deemedio MT, Hedrick MH, Roos KP (2009). Adipose tissue-derived cells improve cardiac function following myocardial infarction.. J Surg Res.

[127] Valina C, Pinkernell K, Song YH, Bai X, Sadat S, Campeau RJ (2007). Intracoronary administration of autologous adipose tissue-derived stem cells improves left ventricular function, perfusion, and remodelling after acute myocardial infarction.. Eur Heart J.

[128] Ogden CL, Carroll MD, McDowell MA, Flegal KM (2007). Obesity among adults in the United States – no statistically significant change since 2003-2004.. NCHS Data Brief.

[129] Mbanya JCN, Motala AA, Sobngwi E, Assah FK, Enoru ST (2010). Diabetes in sub-Saharan Africa.. Lancet.

[130] Mutimura E, Crowther NJ, Stewart AV, Cade WT (2008). HIV and the cardiometabolic syndrome in the developing world: an African perspective.. J Cardiometab Syndr.

[131] Tollman SM, Kahn K, Sartorius B, Collinson MA, Clark SJ, Garenne ML (2008). Implications of mortality transition for primary health care in rural South Africa: a population-based surveillance study.. Lancet.

[132] Walley AJ, Asher JE, Froguel P (2009). The genetic contribution to non-syndromic human obesity.. Nature Rev Genet.

[133] Alvarez-Llamas G, Szalowska E, de Vries MP, Weening D, Landman K, Hoek A (2007). Characterization of the human visceral adipose tissue secretome.. Mol Cell Proteomics.

[134] Hong SJ, Traktuev DO, March KL (2010). Therapeutic potential of adipose-derived stem cells in vascular growth and tissue repair.. Curr Opin Organ Transpl.

[135] Madonna R, De Caterina R (2010). Adipose tissue: a new source for cardiovascular repair.. J Cardiovasc Med (Hagerstown)..

[136] Cherubino M, Marra KG (2009). Adipose-derived stem cells for soft tissue reconstruction.. Regen Med.

[137] De Villiers JA, Houreld N, Abrahamse H (2009). Adipose derived stem cells and smooth muscle cells: implications for regenerative medicine.. Stem Cell Rev.

[138] Taxonera C, Schwartz DA, García-Olmo D (2009). Emerging treatments for complex perianal fistula in Crohn’s disease.. Wld J Gastroenterol.

[139] Wood L, Juritz J, Havemann J, Lund J, Waldmann H, Hale G, Jacobs P (2008). Pediatric immunohematopoietic stem cell transplantation at a tertiary care center in Cape Town.. Hematol Oncol Stem Cell Ther.

